# Sixty-One-Year-Old Female With Metastatic Poorly Differentiated Carcinoma of the Appendix With Omental Metastasis

**DOI:** 10.7759/cureus.8688

**Published:** 2020-06-18

**Authors:** David McNamara, Benjamin Raymond

**Affiliations:** 1 Oncology, United Hospital Center, Bridgeport, USA; 2 General Surgery, United Hospital Center, Bridgeport, USA

**Keywords:** neuroendocrine carcinoma, omental metastasis, metastatic tumors of the omentum, carcinoma of the appendix, appendiceal neuroendocrine tumor, abdominal pain, appendectomy, stage iv, ajcc, enets

## Abstract

Neuroendocrine tumors (NETs) are rare tumors that are often asymptomatic and were once considered benign. A specific subtype that we will dive into in this article is appendiceal neuroendocrine tumors (ANETS). ANETs are the most common tumors located within the appendix. Most often, they present as acute appendicitis and are found incidentally on pathology reports status post appendectomy. The objective of this article is to show that even though most of the ANETs are benign and fully treated via surgery, ANETs still have the potential to become malignant and metastasize. Our patient fits the common features seen in ANETS. She is a middle-aged woman with vague abdominal pain and no clear diagnosis on gastrointestinal (GI) workup. Computed tomography (CT) confirmed appendicitis, and pathology reports status post-surgery confirmed stage IV, pT4, Nx, M1 - poorly differentiated neuroendocrine carcinoma of the appendix with omental metastases.

## Introduction

In 1907, Dr. Siegfried Oberndorfer was the first scientist to characterize the neuroendocrine tumor (NET), which he referred to as “benign carcinomas” and subsequently named them Kazinodes or “carcinoma -like” tumors [1]. From the beginning of his work, there have been a plethora of new and insightful advances in NET research, although most cases lack a focus on the malignant potential of these rare tumors. When the specific location of NETs is appendiceal neuroendocrine tumors (ANETs), they are even rarer but usually have a favorable outcome in most documented cases [2-3]. ANETs are usually asymptomatic and are often found incidentally after the patient is diagnosed with acute appendicitis via abnormal computed tomography (CT), magnetic resonance imaging (MRI), or ultrasound (US) imaging and specimens come back positive post-cholecystectomy. ANETs carry better survival rates (>95%) as compared to all other tumor types located in the appendix [4]. Although these ANETs are seemingly rare and most often benign, there are always exceptions and outliers, in which our patient was found to be.

This article was featured as a poster in the Society of Hospital Medicine - West Virginia Chapter.

## Case presentation

A 61-year-old obese female presented to the gastroenterology clinic for evaluation of nagging abdominal pain that had been present for eight months. She described the pain as sharp, localized to the periumbilical region/left upper quadrant, and lasting for a few minutes to a few hours at a time. The pain occasionally escalates to 10/10 in intensity. She currently has no associated symptoms and denied any aggravating or relieving factors. Past medical history reveals irritable bowel syndrome with alternating periods of diarrhea and constipation, bloating, nausea, well-controlled asthma, and gastroesophageal reflux disease (GERD). She described her diarrhea as watery, occurring three times a day and not present throughout the night. She had no history of fevers, odynophagia, dysphagia, frank hematemesis, hematochezia, or melena. She has an extensive past surgical history, including laparoscopic cholecystectomy, open abdominal hysterectomy with bilateral salpingo-oophorectomy, caesarian sections x 2, and tubal ligation. She was currently taking Prilosec and asthma medications. Family history was insignificant. She had never smoked and denied any frequent alcohol use or illicit drugs.

**Figure 1:**
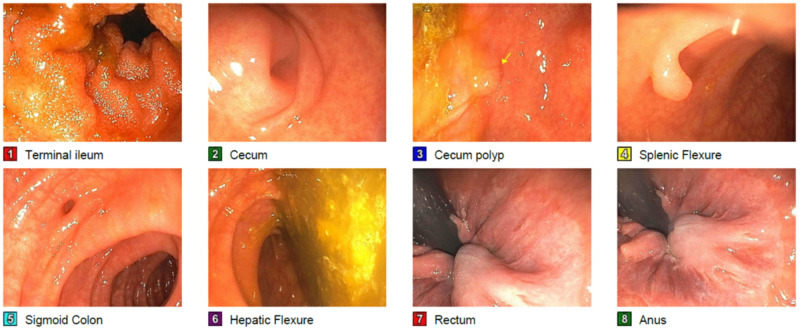
Colonoscopy - The examined portion of the ileum was normal. - One 5 mm polyp in the cecum, removed with a cold snare. Resected and retrieved. - One 5 mm polyp at the splenic flexure, removed with a cold snare. Resected and retrieved. - Diverticulosis in the sigmoid colon. - External hemorrhoids.

**Figure 2:**
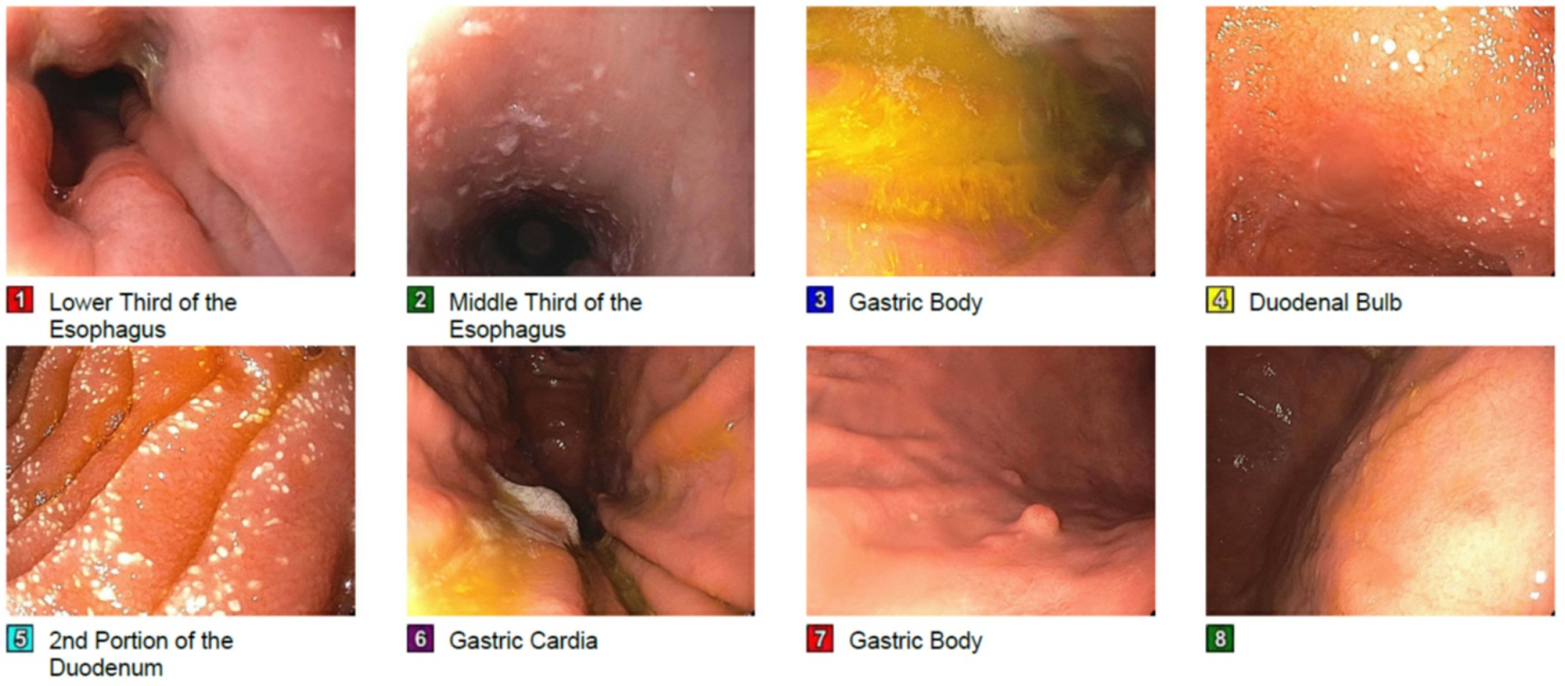
EGD - Z-line irregular. - Erythematous mucosa in the antrum. Biopsied. - A few gastric polyps. Biopsied. - Normal duodenal bulb and 2nd part of the duodenum. Biopsied. EGD: esophagogastroduodenoscopy

**Figure 3:**
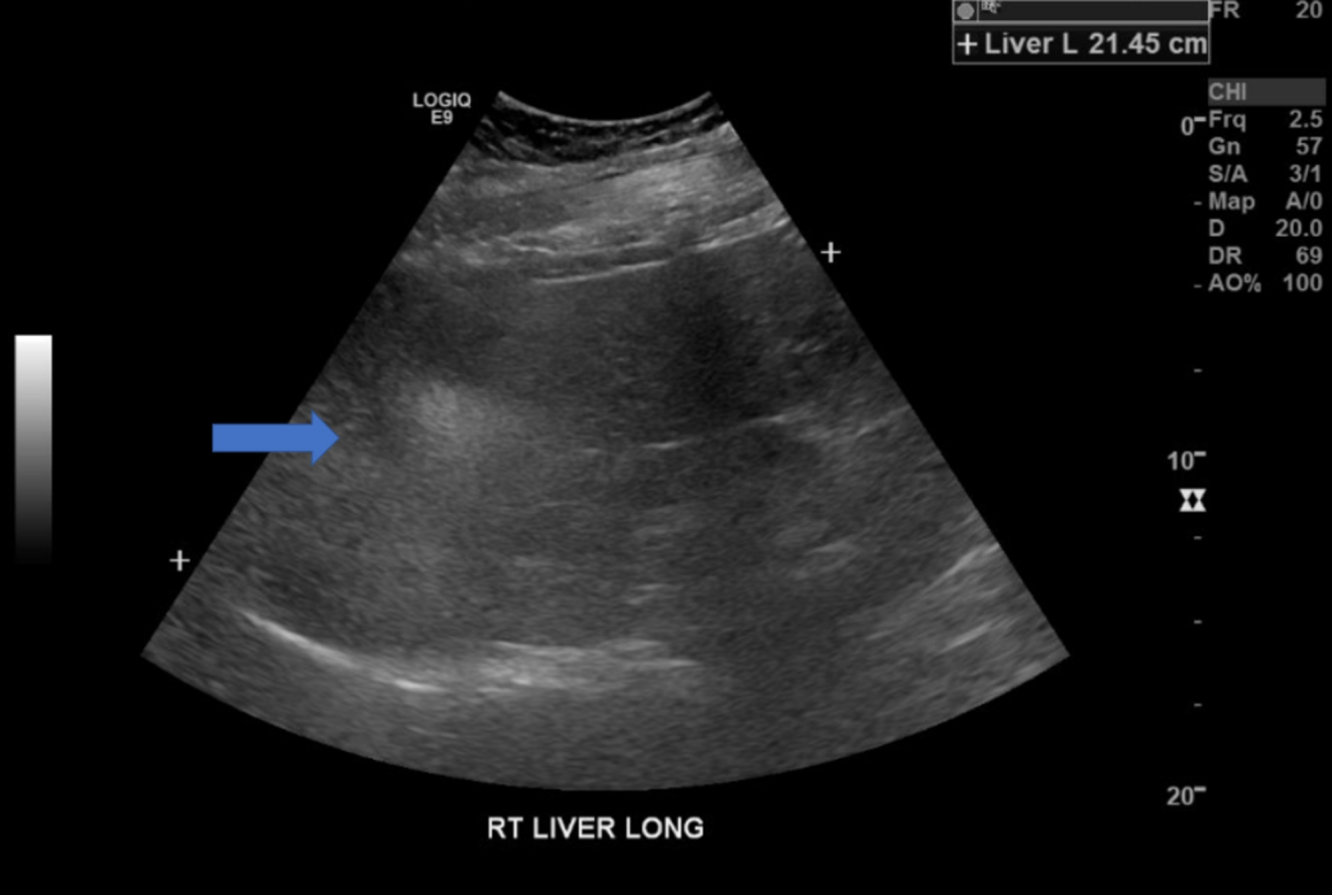
US of liver - Hepatic enlargement with associated fatty infiltration changes. US: ultrasound

**Figure 4:**
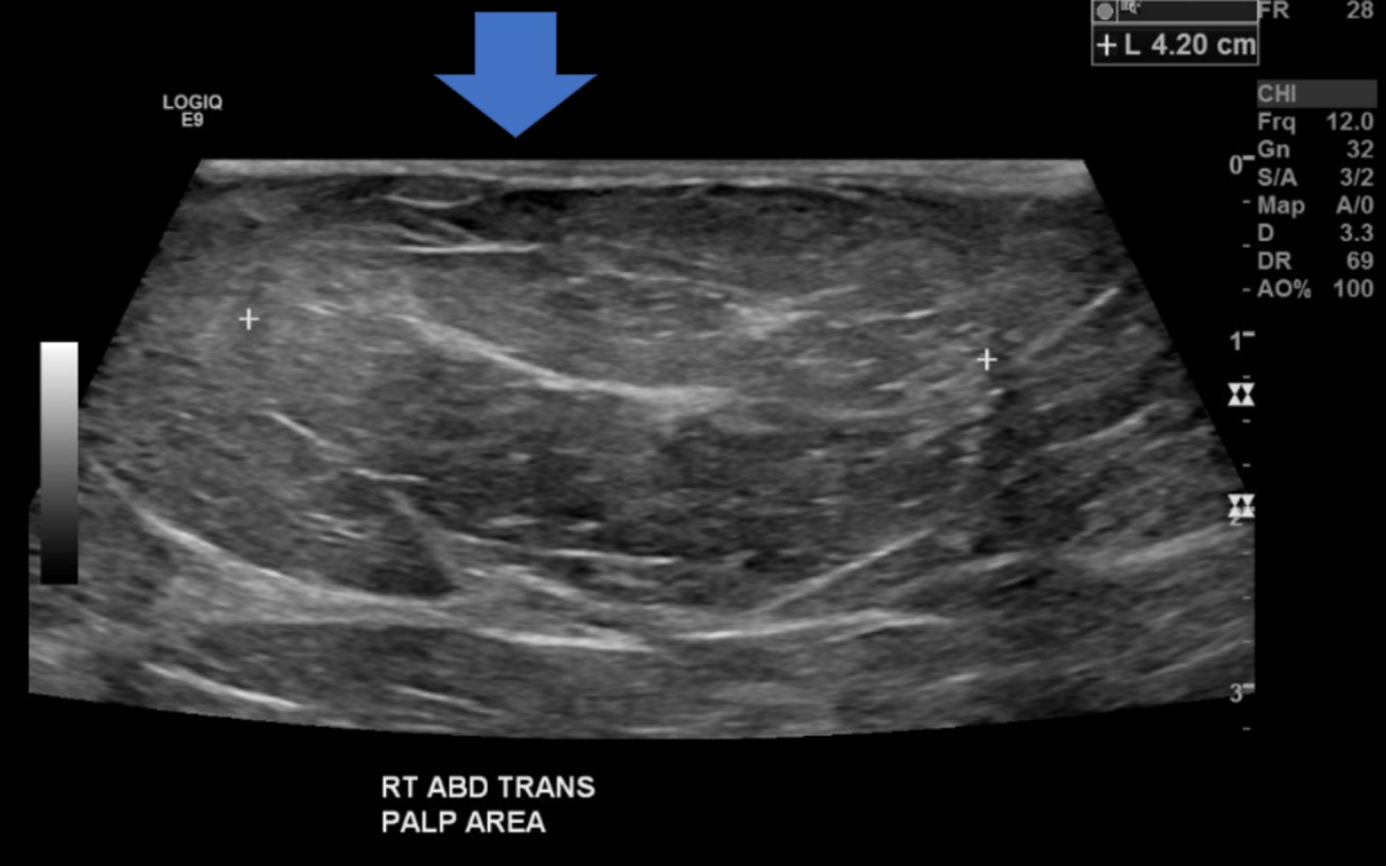
US of abdomen - Suspect fat-containing ventral abdominal wall hernia. US: ultrasound

On physical exam, she appeared chronically ill but in no acute distress. Her abdomen was obese, soft, and tender to deep palpation in the epigastric area. Fullness is noted in the right lateral aspect of the right upper quadrant. No rebound tenderness or guarding was noted. Positive bowel sounds were present in all four quadrants. Labs, including hepatic function, complete blood count (CBC) with differential, and basic metabolic panel (BMP) are all within normal limits (Tables 1-3). She was scheduled for a full GI workup, including an upper esophagogastroduodenoscopy (EGD), colonoscopy, abdominal US, endomysial antibodies, tissue transglutaminase, gliadin antibodies, and serum immunoglobulin A (IgA), which were all within normal limits (Table 4). Within two months, her colonoscopy, EGD, and US were performed. Colonoscopy was unremarkable aside from a few small polyps and diverticula were noted (Figure 1). EGD was largely unremarkable aside from a few gastric polyps and an irregular Z-line (Figure 2). Her abdominal US showed hepatic enlargement with associated fatty infiltration changes status post-cholecystectomy, and a ventral abdominal wall hernia containing abdominal fat (Figures 3-4). She was referred to urology, who stated her pain was of non-urological origin after a full genitourinary (GU) workup.

**Table 1:** Hepatic function panel AST: aspartate aminotransferase; SGOT: serum glutamic-oxaloacetic transaminase; ALT: alanine transaminase

HEPATIC FUNCTION PANEL	
TOTAL PROTEIN	6.5
ALBUMIN	2.8 (L)
BILIRUBIN, TOTAL	1.1
AST (SGOT)	21
ALT (SGPT)	15
ALKALINE PHOSPHATASE	97

**Table 2:** CBC with differential CBC: complete blood count; HGB: hemoglobin; HCT: hematocrit; RBC: red blood cell; MCV: mean corpuscular volume; MCHC: mean corpuscular hemoglobin; concentration; RDQ: reflux disease questionnaire; MPV: mean platelet volume

COMPLETE BLOOD COUNT WITH DIFFERENTIAL	
WBC	3.8
HGB	11.4
HCT	33.8
PLATELET COUNT	206
RBC	3.64
MCV	92.8
MCHC	33.6
MCHC	31.2
RDQ	15
MPV	8.2

**Table 3:** BMP BMP: basic metabolic panel; BUN: blood urea nitrogen

BASIC METABOLIC PANEL	
SODIUM	139
POTASSIUM	4.1
CHLORIDE	108
CARBON DIOXIDE	25
BUN	12
CREATININE	0.82
GLUCOSE	108
ANION GAP	6
BUN/CREAT RATIO	15
ESTIMATED GLOMERULAR FILTRATION RATE	>59
CALCIUM	9.3

**Table 4:** Immunology panel IGA: immunoglobulin A; IGG: immunoglobulin G

IMMUNOLOGY PANEL	
GLIADIN (DEAMIDATED ) ANTIBODY IGA QUALITATIVE	Negative
GLIADIN (DEAMIDATED) ANTIBODY IGA QUANTITATIVE	<0.2
GLIADIN (DEAMIDATED) ANTIBODY IGG QUALITATIVE	Negative
GLIADIN (DEAMIDATED) ANTIBODY IGG QUANTITATIVE	<0.4
IMMUNOGLOBULIN A	194
TISSUE TRANSGLUTAMINASE ANTIBODIES IGA QUALITATIVE	Negative
TISSUE TRANSGLUTAMINASE ANTIBODIES IGA QUANTITATIVE	<0.5
TISSUE TRANSGLUTAMINASE ANTIBODIES IGG QUALITATIVE	Negative
TISSUE TRANSGLUTAMINASE ANTIBODIES IGG QUANTITATIVE	<0.8
ENDOMYSIAL ANTIBODIES	Negative

**Figure 5:**
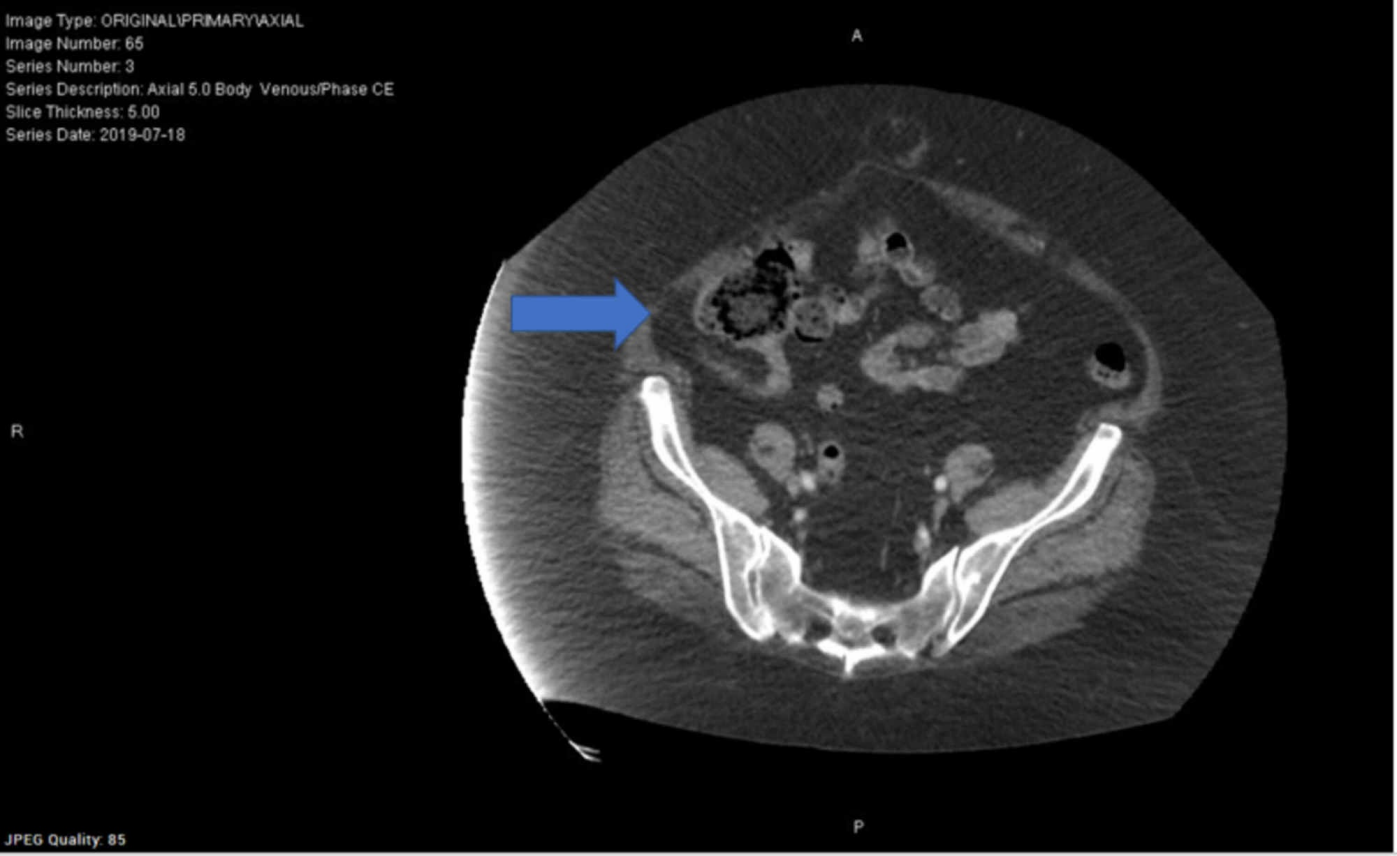
CT abdomen - Thickened appendix with possible mild inflammation. Acute appendicitis is suspected. - Umbilical hernia containing fat. CT: computed tomography

**Figure 6:**
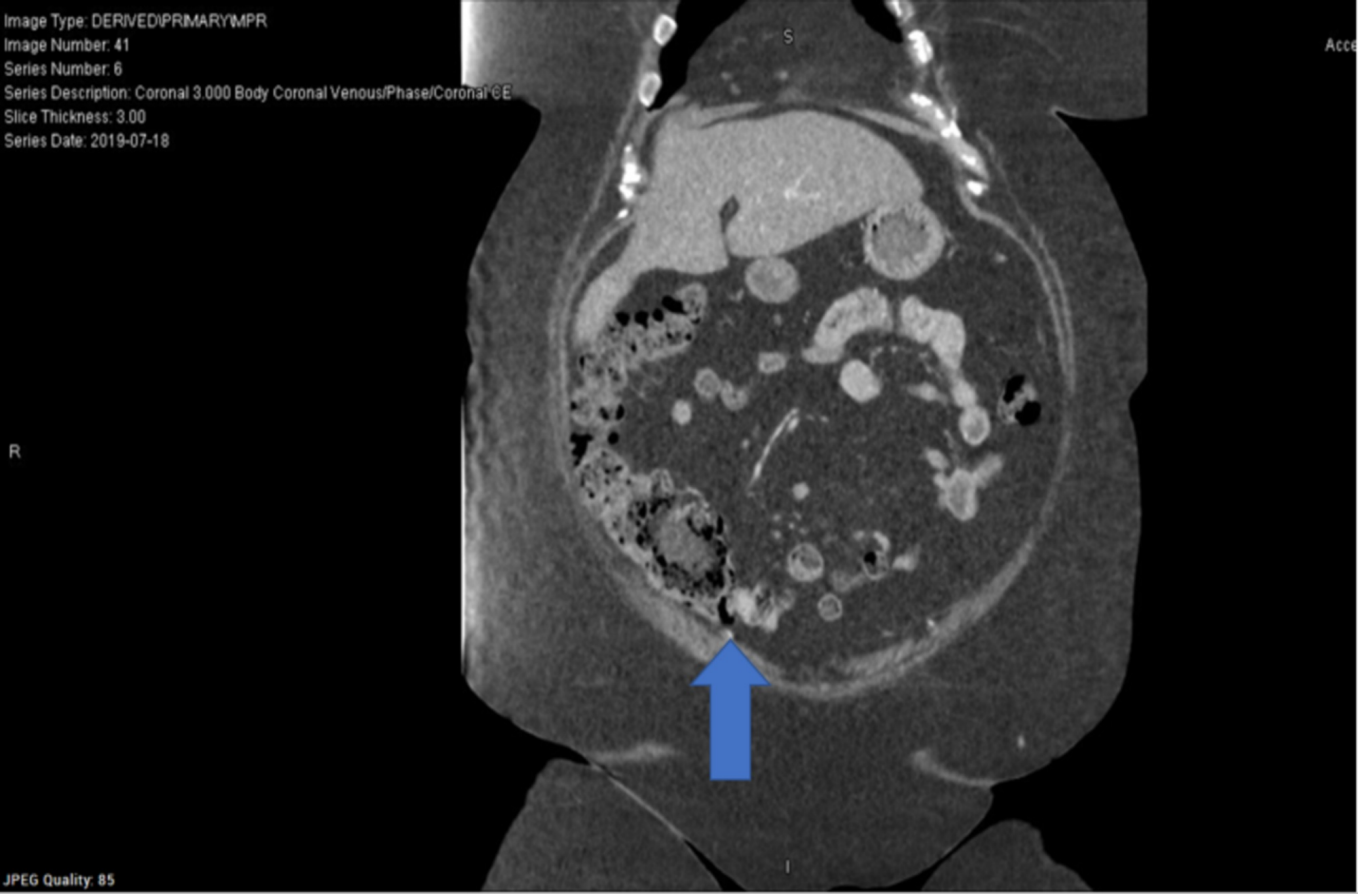
CT abdomen - Thickened appendix with possible mild inflammation. Acute appendicitis is suspected. - Umbilical hernia containing fat. CT: computed tomography

**Figure 7:**
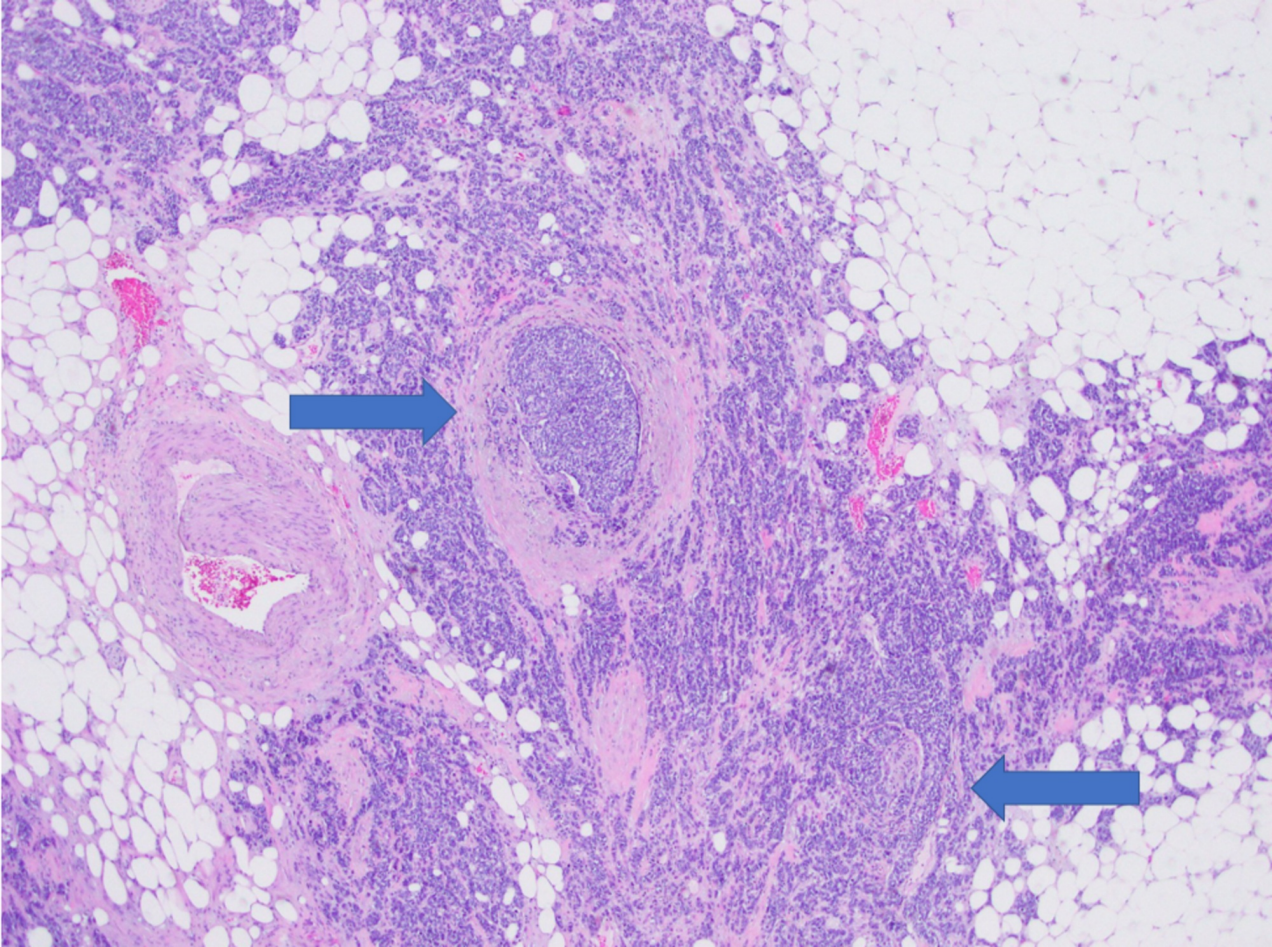
Positive for lymphovascular and perineural invasion

**Figure 8:**
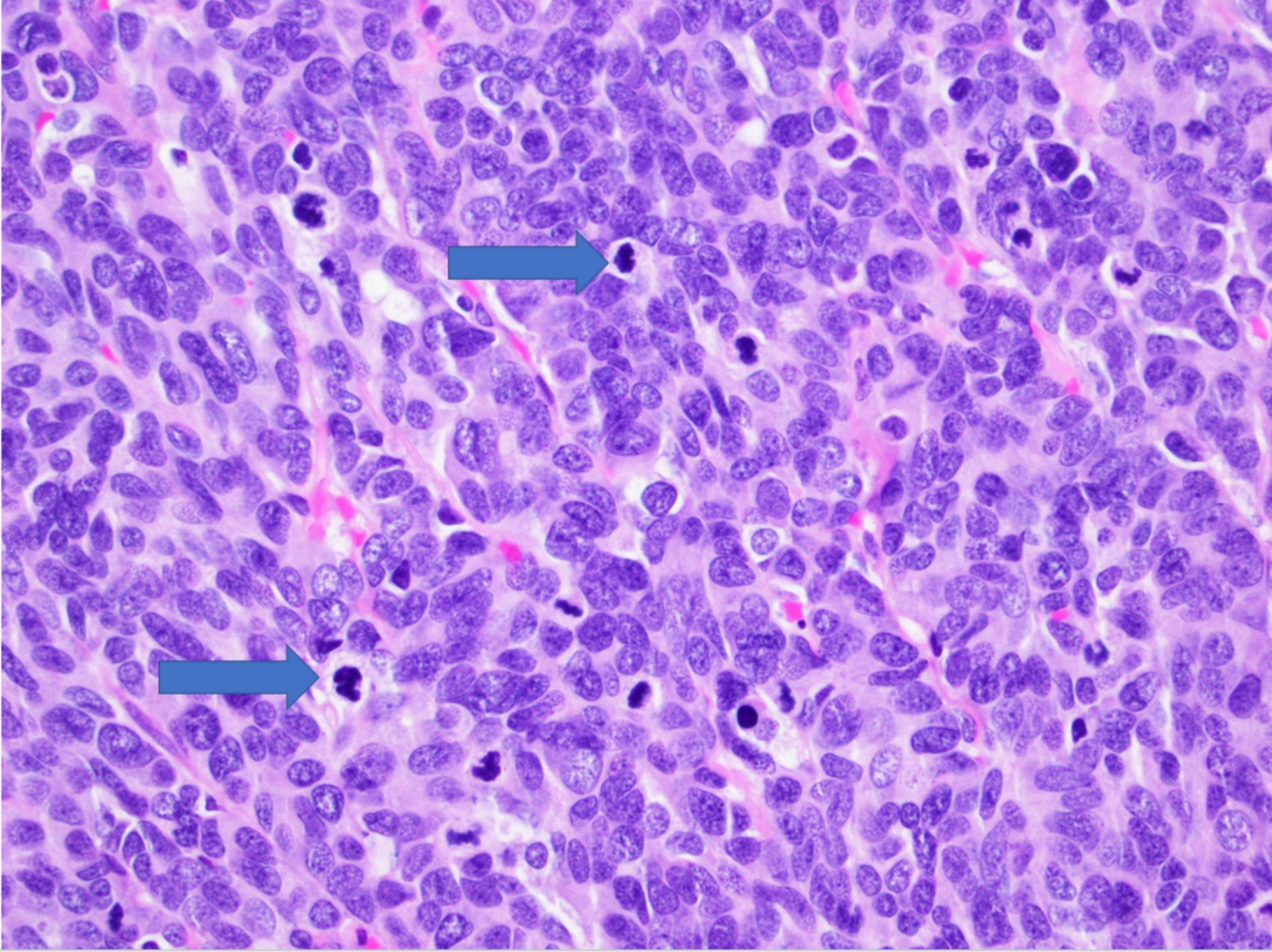
High power view Large amount of mitotic bodies seen.

**Figure 9:**
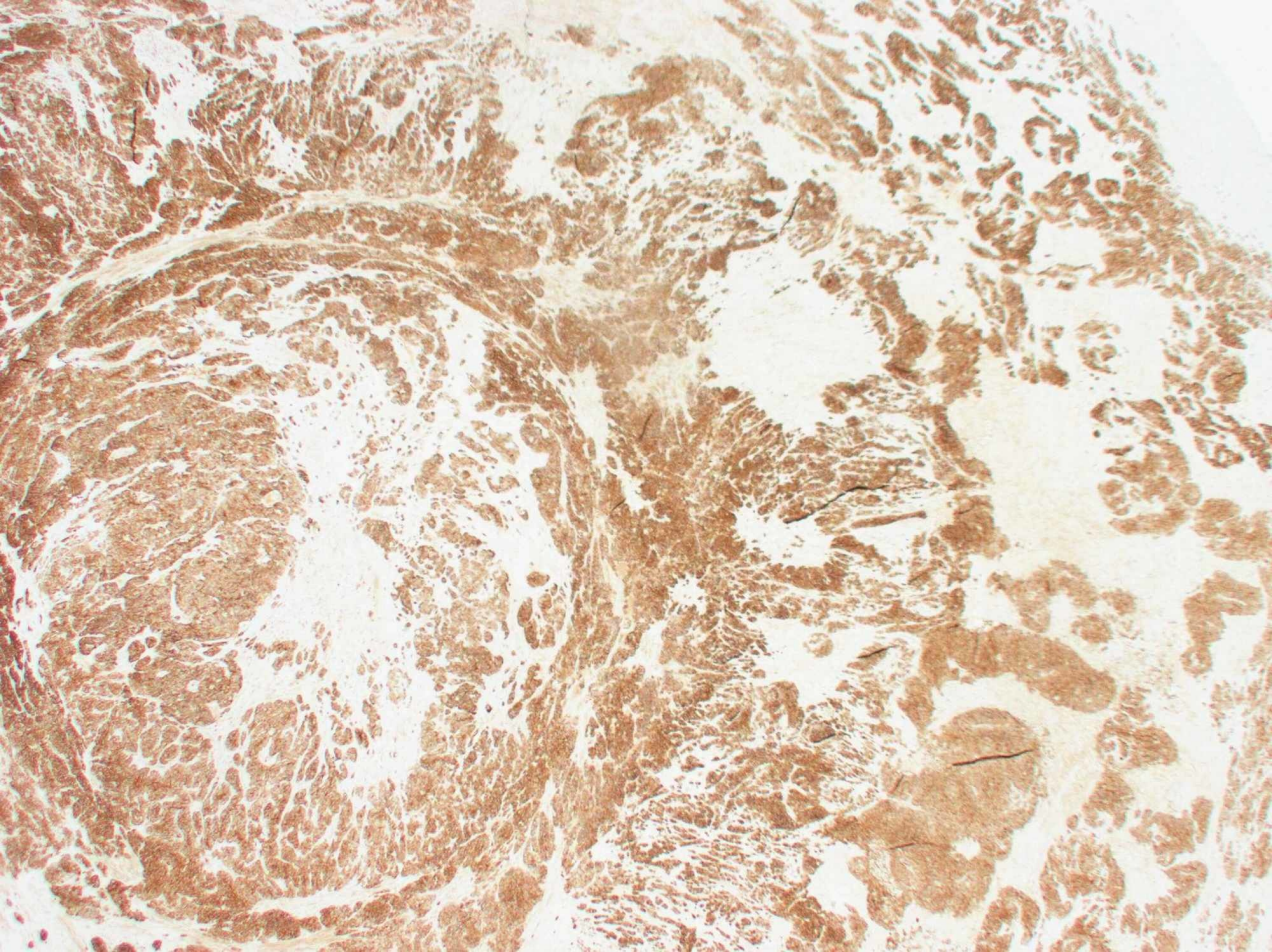
Positive for CD56 staining

Approximately three months later, she reported to the emergency department (ED) for abdominal pain, where a CT abdomen was performed and showed a dilated appendix with inflammatory stranding concerning for acute appendicitis as well as a supra-umbilical incisional hernia containing no loops of the bowel (Figures 5-6). During surgery, there was a vast amount of adhesions secondary to dense adhesion disease, which were ligated x 30 minutes. The surgeon also ligated some abdominal fat within the hernia defect as necessary to reduce the hernia. All ligated specimens, as well as the appendix, were sent to pathology. The pathology report confirmed an incarcerated hernia sac and appendix specimens showing metastatic, poorly differentiated neuroendocrine carcinoma. The tumor present measured 7.4 cm and was a pT4a grade 3 tumor, positive for lymphovascular and perineuronal invasion (Figure 7). The proximal margin was positive for invasive carcinoma. A mitotic rate of 50 mitoses/hpf was found; Ki67 56% for a final report of Stage IV: pT4, Nx, M1 poorly differentiated neuroendocrine carcinoma of the appendix with omental metastases (Figure 8). Immunostains were positive for AE1/3, CD56, CK20, TTF-1, and synaptophysin and were negative for MCPyV (Figure 9).

**Figure 10:**
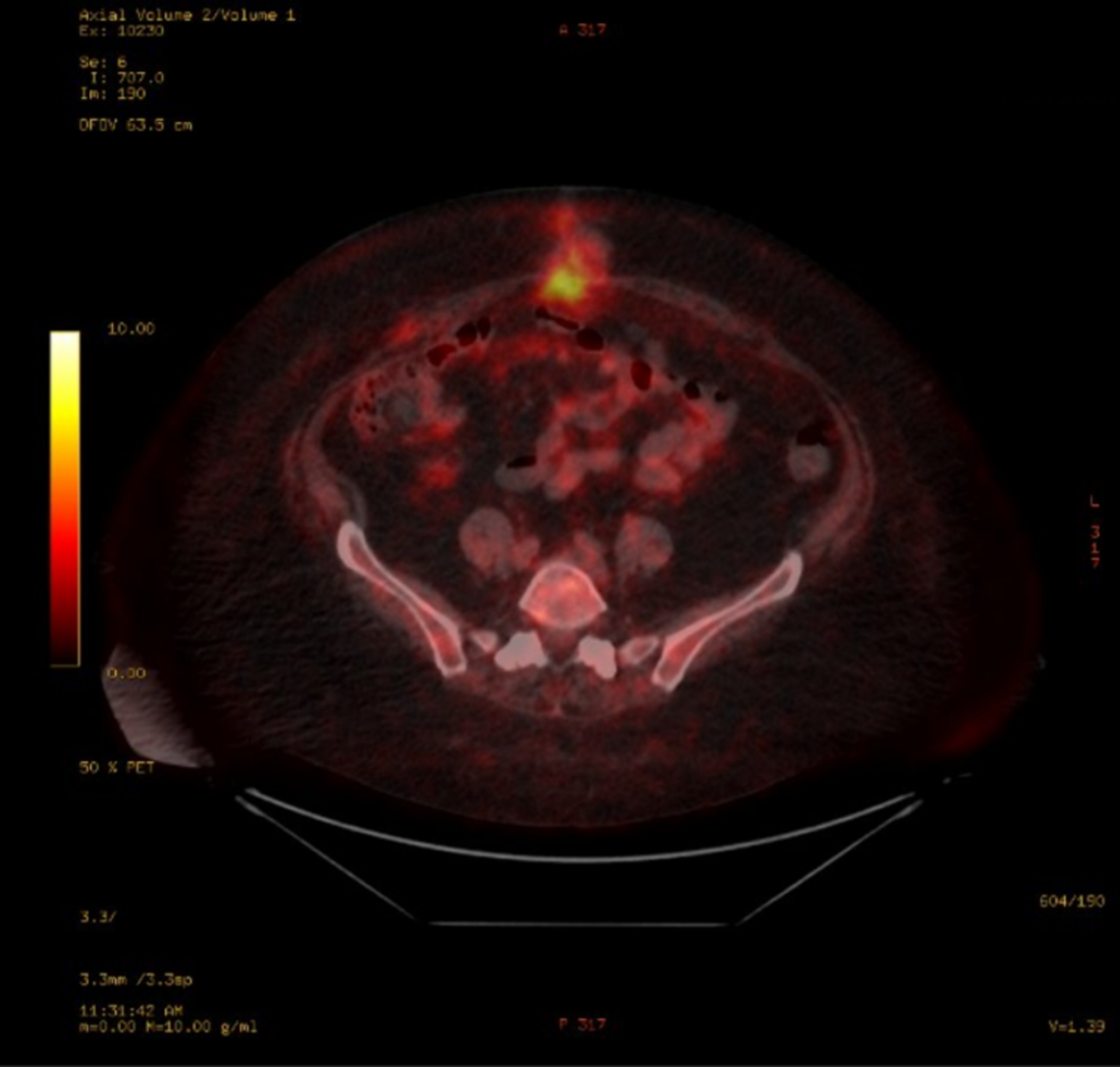
PET-CT axial - No definite evidence for metastatic disease. Patient is status post appendectomy. PET-CT: positron emission tomography-computed tomography

**Figure 11:**
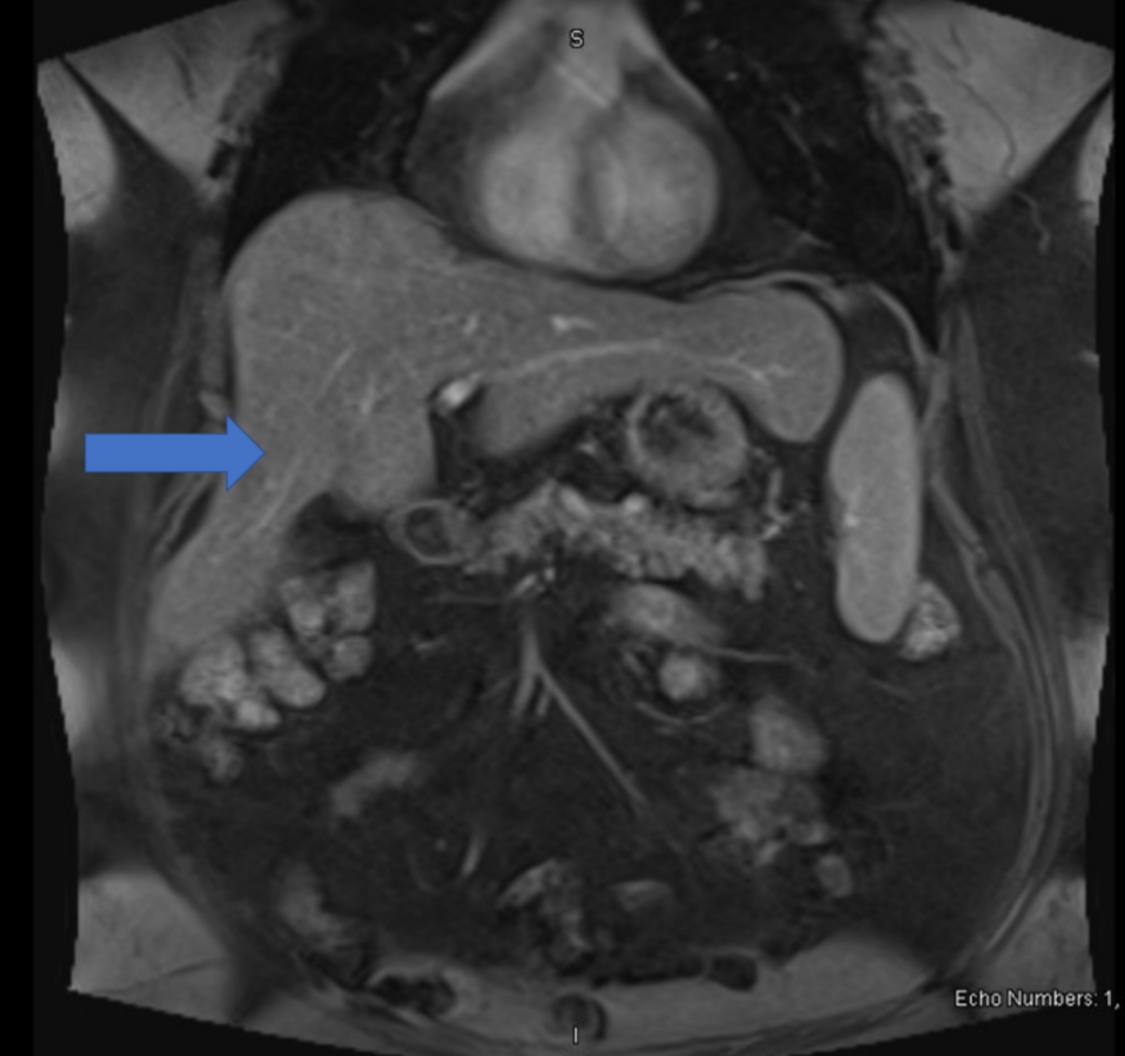
MRI abdomen - Hepatic enlargement with associated fatty infiltration change. - Postoperative changes status post cholecystectomy without intrahepatic or extra-hepatic biliary duct dilatation. MRI: magnetic resonance imaging

**Figure 12:**
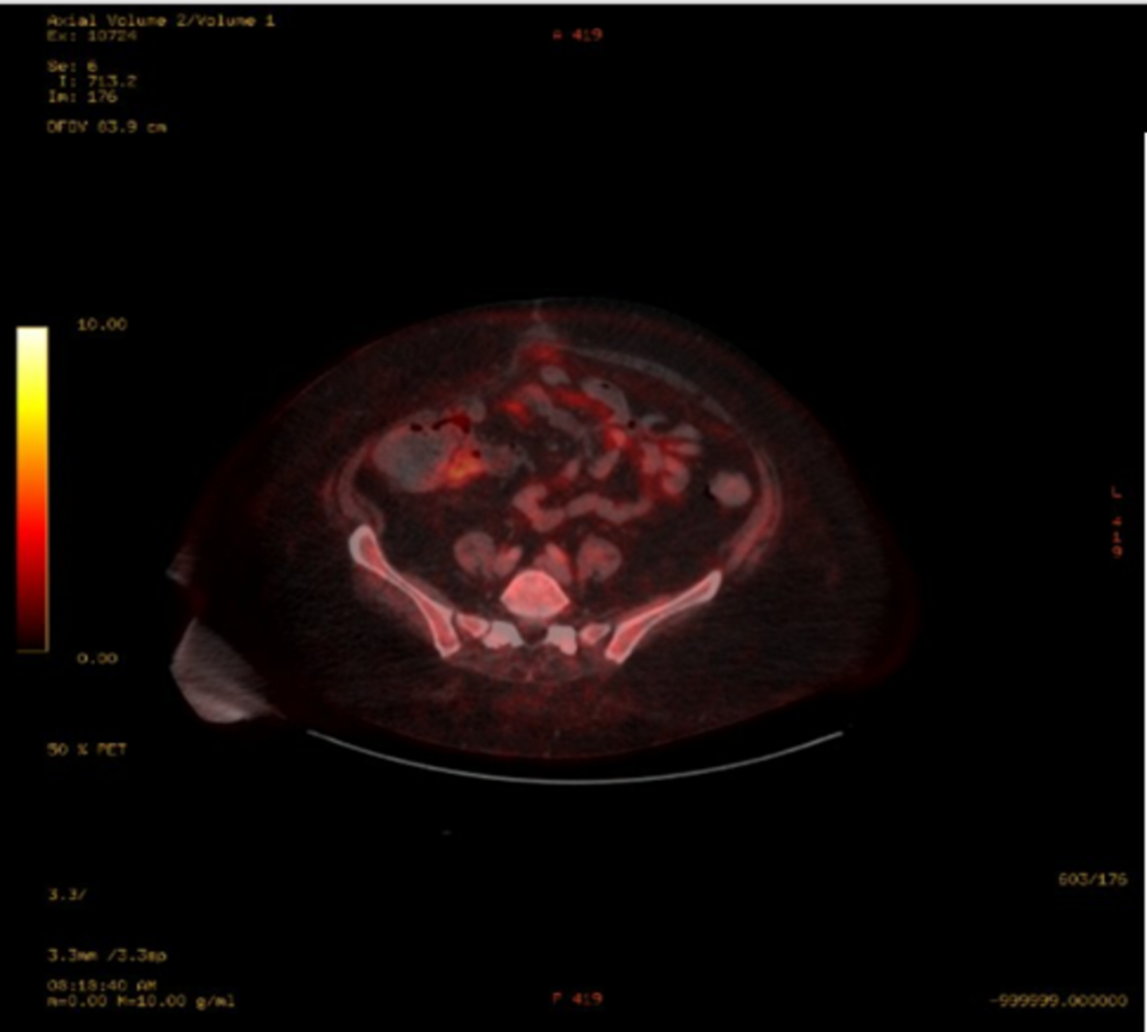
Repeat PET-CT - No evidence of recurrent malignancy or metastatic disease. PET-CT: positron emission tomography-computed tomography

**Figure 13:**
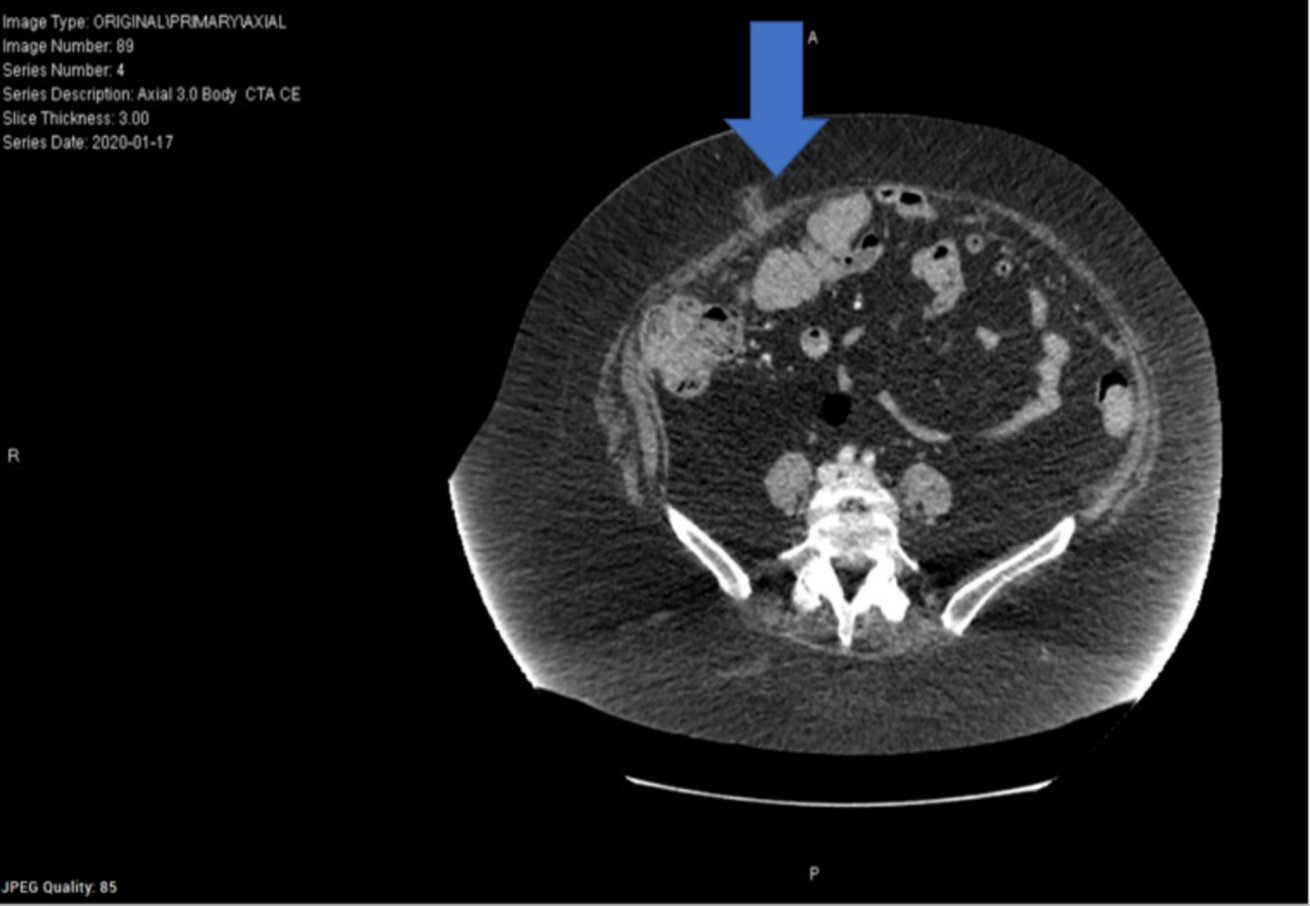
Repeat CT abdomen - Nodularity in the omentum particularly in the lower abdomen concerning for omental metastasis. Structures anterior to the falciform ligament within the peritoneal fat. These findings are increased/new from prior imaging. There is a concern these may represent omental metastasis. CT: computed tomography

She was subsequently seen by an oncologist who performed a positron emission tomography-computed tomography (PET-CT) scan that showed no definite evidence for metastatic disease s/p appendectomy (Figure 10). The patient's initial plan was to start carboplatin AUC5 for a total dose of 665MG and etoposide 100MG/M2 for a total dose of 246MG x six cycles and refer back to general surgery for port placement to begin the chemotherapy. Every three weeks, the patient was scheduled for a chemotherapy cycle but, unfortunately, due to adverse side effects and constant ED visits, she stopped treatment after four cycles. She continued to complain of intractable abdominal pain throughout her visits, thus an abdominal MRI and repeat PET-CT were ordered, which both showed no evidence of recurrent malignancy or metastatic disease (Figures 11-12). Her intractable abdominal pain never subsided, so she was subsequently referred to an oncological surgeon who proceeded with an exploratory laparoscopy. The laparoscopy showed anterior abdominal wall and omental peritoneal disease. Biopsies taken during the procedure confirmed a high-grade neuroendocrine tumor consistent with prior diagnosis suggesting microscopic disease that was undetectable by imaging. Approximately one month after the exploratory laparoscopy, she returned to the ED where she had a repeat CT abdomen. Upon comparing to previous scans, she, unfortunately, showed worsening nodularity, particularly in the lower abdomen concerning for omental metastasis, signifying recurrent disease (Figure 13). Given these results, treatment with Ipilimumab 1 mg/kg was recommended as well as nivolumab 240 mg flat dose, with a second opinion from Johns Hopkins oncology confirming this plan.

## Discussion

During a 16-year period, 7,970 appendectomies were performed in one hospital, and of those, 74 patients (0.9%) presented with appendiceal tumors (42 carcinoid, 12 benign, and 20 malignant). Primary malignant tumors of the appendix were found in 0.1% of all the appendectomies [2]. After small bowel and rectum malignant NETS, malignant appendiceal NETs represent the third most common malignant neuroendocrine neoplasms of the gastrointestinal tract with an annual incidence of 0.63 cases per million of the general population [3]. ANETs are most often found at the distal tip of the appendix, as they are derived from a subepithelial cell line [5]. There is a prevalence in middle-aged women around the fifth decade of life, although some believe this could be because women have more diagnostic laparoscopic procedures for atypical lower abdominal pain [6]. There is no preference for race [7]. ANETs are most often asymptomatic, although they are usually found incidentally when the patient presents with symptoms of acute appendicitis via CT scan and pathology specimens reveals ANET postoperatively. ANETs can show a diffuse infiltrative pattern that manifests as a diffuse mural thickening on cross-sectional imaging [8].

Surgery is largely curative, with most patients living a long and fruitful life afterward. Surgery guidelines are relatively straightforward, although the classification is a little more debated over. As a general rule, ANETs <1 cm hardly ever metastasize and are treated by appendectomy alone while ANETs >2 cm call for a right hemicolectomy due to the high risk of metastasis [5]. Tumors >1 cm but <2cm should receive a treatment plan, including an appendectomy followed by scheduled follow-up for five years [3]. According to the European Neuroendocrine Tumor Society (ENETS), tumors >1 cm but <2 cm, with features indicating a higher risk of lymph node dissemination, such as mesoappendiceal invasion >3 mm, localization in the base of the appendix, vascular infiltration, or intermediate differentiation should be watched very closely and have yearly follow-ups [7,9-10].

Due to the misconceptions that NETs were once proposed to be nonmalignant, as Dr. Oberndorfer originally believed, there have been two new classification schemes, the first was presented by ENETS in 2006 and the other by the American Joint Committee on Cancer (AJCC), which appeared in AJCC 8th edition in 2010 [11-12]. These classification schemes use the invasion of the mesoappendix and require the inclusion of more criteria to define the selection of surgical treatment of tumors between 1 cm and 2 cm. Thus, along with the invasion of the mesoappendix and size of the tumor, other factors such as vascular invasion, ki67 index, mitotic index, and tumor location should be considered at the time of classification, for a better selection of the treatment and prognostic evaluation [11]. The World Health Organization (WHO) also developed a classification for NETs within the gastrointestinal tract. Their classification considers the well-defined pathological features such as size, lymphovascular invasion, mitotic counts, ki-67 index, invasion of adjacent organs, presence of metastases, and functional status [13].

Our patient lines up with the typical demographics and clinical presentation seen in ANETs. She is a 61-year-old female with vague abdominal pain who had a full GI and GU workup with no definitive diagnosis. She eventually obtained a CT abdomen, which came back as acute appendicitis. While in surgery for the laparoscopic cholecystectomy, it was very fortunate that the surgeon removed much of the abdominal fat in the hernia sac, which came back positive for poorly differentiated neuroendocrine carcinoma. The appendix was positive as well and had a final pathology report of stage IV: pT4, Nx, M1 poorly differentiated neuroendocrine carcinoma of the appendix with omental metastases. Due to the metastases, large tumor size, high mitotic rate, and Ki67%, surgery was not the treatment option but rather immunotherapy medications. Unfortunately, her disease continues to cause her significant pain even with the placement of a hydromorphone PCA pump. The patient is in good spirits and continues to be optimistic about treatments, although she understands the grave prognosis that she is faced with.

## Conclusions

Neuroendocrine tumors (NETs) were once believed to be non-malignant and of little clinical significance. Dr. Siegfried Oberndorfer’s research into carcinoid tumors sparked a new revolution for NETs. Appendiceal NETs (ANETS) happen to be the most common tumor found in the appendix overall. Over the years, the malignancy rate has been very low indeed, but as in our patient's case, statistical outliers do exist and need to be explored. The purpose of this case report is to show that ANETS can be a malignant tumor with the potential for metastases and should not be taken lightly. Whether ANETS are malignant or not, they usually tend to be asymptomatic. ANETS often present in middle-aged women and are most often found when a patient presents with symptoms congruent with acute appendicitis. CT or other imaging confirms the diagnosis of acute appendicitis and an appendectomy is earned. It is not until postoperative pathological reports come back positive for ANET that the neoplasm is finally known. Thanks to the WHO, ENETS, and AJCC, there are specified guidelines on how to classify each type of ANET and what the best treatment and follow-up is. Surgery is largely curative if the ANET is caught before it can grow too large or has the opportunity to metastasize. In the case of metastasis, as in our patient, immunotherapy can be initiated if the patient so wishes. Close follow-up should be applied for all types of ANETS.
